# FABP7 is a potential biomarker to predict response to neoadjuvant chemotherapy for breast cancer

**DOI:** 10.1186/s12935-020-01656-3

**Published:** 2020-11-23

**Authors:** Qin Xie, Ying-sheng Xiao, Shi-cheng Jia, Jie-xuan Zheng, Zhen-chao Du, Yi-chun Chen, Mu-tong Chen, Yuan-ke Liang, Hao-yu Lin, De Zeng

**Affiliations:** 1 Department of Medical Oncology, The Cancer Hospital of Shantou University Medical College, 7 Raoping Road, Shantou, 515031 People’s Republic of China; 2 Guangdong Provincial Key Laboratory for Breast Cancer Diagnosis and Treatment, Cancer Hospital of Shantou University Medical College, Shantou, 515031 Guangdong People’s Republic of China; 3 Department of Thyroid Surgery, Shantou Central Hospital, 114 Waima Road, Shantou, 515031 People’s Republic of China; 4 Shantou University Medical College, Shantou, 515000 People’s Republic of China; 5 Department of Thyroid and Breast Surgery, The First Affiliated Hospital of Shantou University Medical College, 57 Changping Road, Shantou, 515041 People’s Republic of China

**Keywords:** Fatty acid binding protein-7 (FABP7), Chemosensitivity, Prognosis, Breast cancer

## Abstract

**Background:**

Early prediction of response to neoadjuvant chemotherapy (NAC) is critical in choosing appropriate chemotherapeutic regimen for patients with locally advanced breast cancer. Herein, we sought to identify potential biomarkers to predict the response to neoadjuvant chemotherapy for breast cancer patients.

**Methods:**

Three genomic profiles acquired by microarray analysis from subjects with or without residual tumors after NAC downloaded from the GEO database were used to screen the differentially expressed genes (DEGs). An array of public databases, including ONCOMINE, cBioportal, Breast Cancer Gene Expression Miner v4.0, and the Kaplan Meir-plotter, etc., were used to evaluate the potential functions, related signaling pathway, as well as prognostic values of FABP7 in breast cancer. Anti-cancer drug sensitivity assay, real-time PCR, flow cytometry and western-blotting assays were used to investigate the function of FABP7 in breast cancer cells and examine the relevant mechanism.

**Results:**

Two differentially expressed genes, including FABP7 and ESR1, were identified to be potential indicators of response to anthracycline and taxanes for breast cancer. FABP7 was associated with better chemotherapeutic response, while ESR1 was associated with poorer chemotherapeutic effectiveness. Generally, the expression of FABP7 was significantly lower in breast cancer than normal tissue samples. FABP7 mainly high expressed in ER-negative breast tumor and might regulate cell cycle to enhance chemosensitivity. Moreover, elevated FABP7 expression increased the percentage of cells at both S and G2/M phase in MDA-MB-231-ADR cells, and decreased the percentage of cells at G0/G1 phase, as compared to control group. Western-blotting results showed that elevated FABP7 expression could increase Skp2 expression, while decrease Cdh1 and p27kip1 expression in MDA-MB-231-ADR cells. In addition, FABP7 was correlated to longer recurrence-free survival (RFS) in BC patients with ER-negative subtype of BC treated with chemotherapy.

**Conclusion:**

FABP7 is a potential favorable biomarker and predicts better response to NAC in breast cancer patients. Future study on the predictive value and detail molecular mechanisms of FABP7 in contribution to chemosensitivity in breast cancer is warranted.

## Background

Among female, breast cancer is the most commonly diagnosed cancer across the world and is the leading cause of cancer-related death, accounting for about 11.6% of all cancer mortality [1]. Resistance to chemotherapy in adjuvant settings remains the major culprit of treatment failure for this deadly disease after surgery and radiation therapy [2, 3].

In the past few decades, the use of adjuvant systemic therapy, in addition to surgery, has significantly reduced local relapse and improved survival of patients with breast cancer [4]. Neoadjuvant chemotherapy (NAC), defined as the administration of chemotherapeutic agents before surgery, is a treatment strategy to decrease the extent and size of locally advanced tumors, with purpose of facilitating breast-conserving surgery, rendering locally advanced cancers operable, as well as eliminating occult distant metastases [5, 6].

However, neoadjuvant chemotherapy might be a double-edged sword, since it can be efficient in shrinking the tumor volume, or it could be ineffective and the patients merely suffer from the toxicity and side effects [7]. Early prediction of the possible response from NAC is a critical step for determining whether a current combination should be adopted, or changed to another regimen [8]. In recent years, there is growing interest in identifying the biomarkers that could predict the effectiveness of neoadjuvant chemotherapy for breast cancer [9, 10].

For instance, Jie Li and colleagues reported that higher level of ALDH1 was correlated to poorer responses to NAC in breast cancer patients [11]. Study by Wang and colleagues demonstrated that determination of MMP-9 expression in tumor tissues could help identify triple-negative breast cancer patients who will respond to NAC [12]. Moreover, researchers have found that treatments targeting at key molecules in signaling pathways, including AKT/pERK and FasR/FasL pathways, could enhance the sensitivity of breast cancer to chemotherapy [13, 14].

Currently, robust biomarkers to predict the success of neoadjuvant chemotherapy in breast cancer remains limited. The aim of the present study is to identify potential biomarker that could predict the response to neoadjuvant chemotherapy in patients with breast cancer. Herein, through comprehensive analysis in a series of datasets from multiple public databases, such as GEO, ONCOMINE, cBioportal, and bc-GenExMiner v4.0, we demonstrated that FABP7 was negatively associated with the expression of ESR1, and might better predict response to NAC in patients with ER-negative breast cancer.

## Materials and methods

### Analysis of differentially expressed genes (DEGs) in breast tumors after NAC

Three genomic profiles of breast cancer, including GSE21997, GSE32646, and GSE25055, acquired from the NCBI-GEO database (http://www.ncbi.nlm.nih.gov/geo/) were used to screen the genes that differentially expressed in breast cancer patients with or without residual tumors after NAC. The platforms of GSE21997, GSE32646, and GSE25055 are GPL7504 Agilent Axon scanner UNC custom 4X44K without Virus, GPL570 [HG-U133_Plus_2] Human Genome Affymetrix U133 Plus 2.0 Array, and GPL96 [HG-U133A] Human Genome Affymetrix U133A Array, respectively.

Differentially expressed genes (DEGs) between Pathologic Complete Response (PCR) and non- Pathologic Complete Response (nPCR) patients were screened using GEO2R in GEO database. The genes were regarded to be DEGs if |log 2-Fold Change| ≥ 2 and *p* < 0.05, and the differential expression level of DEGs was drawn as volcano plot. The intersection among the DEGs of three expression profiles was determined using Venn diagram online bioinformatics tool (http://bioinformatics.psb.ugent.be/webtools/Venn/). A workflow chart of this study was shown in Additional file 1: Fig. S1.

### Identification of the expression pattern of FABP7 in breast cancer

The ONCOMINE database, including a variety of breast cancer datasets, was used to compare the mRNA levels of FABP7 and ESR1 in breast cancer (BC) tissues versus normal breast tissues, respectively. In this study, the Paired Student’s *t* test was used for paired and between-group comparison, and a fold-change > 2 with a *p*-value of < 1E−4 was defined as clinically significant. The Breast Cancer Gene-Expression Miner v4.0 database (http://bcgenex.centregauducheau.fr/BC-GEM/GEM-requete.php) was utilized to analyze the association between mRNA levels of FABP7 and specific clinicopathological features of BC, including ESR1 and different molecular subtypes.

### Prognostic value analysis of FABP7 in breast cancer patients

The association between FABP7 mRNA level and survival outcomes of breast cancer patients was evaluated by Kaplan–Meier plotter (http://kmplot.com), which is an online public database that includes 5143 breast cases [15]. The hazard ratio (HR) and log rank *p*-value was displayed on the webpage. The database divides all patients into different molecular subtypes according to the Sorlie’s subtypes and the long-rank tests was used to obtain the hazard ratio (HR) and *p*-value.

### Cell lines and cell culture

MDA-MB-231 was purchased from the American Type Culture Collection (ATCC), MDA-MB-231-ADR was purchased from Shanghai Chunshi Biotechnology co. LTD. The MDA-MB-231 cells were cultured in DMEM containing 10% FBS (Thermo Fisher Scientific, Waltham, MA, USA), while the MDA-MB-231-ADR was cultured in L15 containing 10% fetal bovine serum (FBS). All the cells were cultured in a humidified 5% CO_2_ incubator at 37 °C.

### Plasmids, small interfering RNA, and transfection

The empty vector pCMV and pCMV-FABP7 plasmids were purchased from Yi Qiao Shen Zhou Science and Technology Ltd (Beijing, China). Small interfering RNA (siRNA) were purchased from GenePharma Company (Suzhou, China). Transfection was performed using Lipofectamine 3000 and P3000 (Life Technology, NY, USA), according to the manufacturer’s protocol.

### Western blotting assay

Cells were lysed in RIPA buffer containing protease inhibitors to extract protein from the cell lines, then the protein was separated by 12% SDS-PAGE and transferred to PVDF membrane. Next, the membrane was probed with specific primary antibodies (Table 1), and then cultured for 1 h with appropriate secondary antibodies. Finally, the electrochemiluminescence was used to detect the expression of protein.

**Table 1:** Antibodies used in this study

Antibody	Cat.#	Company	Concentration species
FABP7	D8N3N	Gene biotechnology international trade (Shanghai)	1:1000, rabbit
ESRα	D6R2W	CST	1:2000, rabbit
Anti-Cdh1	DH01	Calbiochem(La Jolla, CA, USA)	1:2000, mouse
Abti-p27kip1	SC-56338	Santa Cruz Biotechnology Inc. (Santa Cruz, CA, USA)	1:2000, mouse
Anti-Skp2	SC-7164	Santa Cruz Biotechnology Inc.	1:2000, rabbit
Anti βactin	8H10D10	CST	1:2000, mouse

### Real-time PCR(RT-PCR) assay

TRIzol reagent (Thermo Fisher Scientific) was used to extract the total RNA. The RNA was then reverse-transcribed into cDNA using the PrimeScript™ RT Reagent Kit (Takara Bio Inc, Dalian, China). Primer sequences for real-time PCR were listed and shown in Table 2. Then, the mRNA levels of FABP7 were analyzed according to the manufacturer’s instructions.

**Table 2:** Primers used in real-time PCR

Gene	Forward primer	Reverse primer
FABP7	5′-CCAGCTGGGAGAAGAGTTTG-3′	5′-CTCATAGTGGCGAACAGCAA-3′
ESRα	5′-TGCTTCAGGCTACCATTATGGA-3′	5′-TGGCTGGACACATATAGTCGTT-3′
βactin	5′-AGCGAGCATCCCCCAAAGTT-3′	5′-GGGCACGAAGGCTCATCATT-3′

### Anti-cancer drug sensitivity assay

We seeded cells into 96-well plates at a density of 8 × 10^3^ cells/well. The plates were added with doxorubicin, ranging from 0.001 to 10 µmol/L after cell adhesion. After incubation for 48 h, the cell viability was measured with Cell Counting Kit-8 (CCK-8, Dojindo, Japan). Then, we used the spectrophotometer (Thermo) to measure the absorbance of each well at 450 nm. Finally, the GraphPad Prism5 was used to calculate the IC50.

### Cell cycle assay

Cells were synchronized using serum-free medium for 24 h. Then, the cells were trypsinized, washed with PBS, and fixed overnight with 75% ethanol. The next day, the cells were stained with 500 µL of propidium iodide for 30 min in the dark. Finally, the DNA content was measured using BD flow cytometer and the data were analyzed using the FlowJo 7.6 software.

### Statistical analysis

All the statistical analysis in the study was performed by using the Statistical Product and Service Solutions (SPSS) version 23.0. The paired and between-group comparison analysis were performed by using Student’s t-test. Two-sided *p*-value of less than 0.05 was considered statistically significant.

## Results

### FABP7 and ESR1 are differentially expressed in breast cancer cases received NAC

**Fig. 1:**
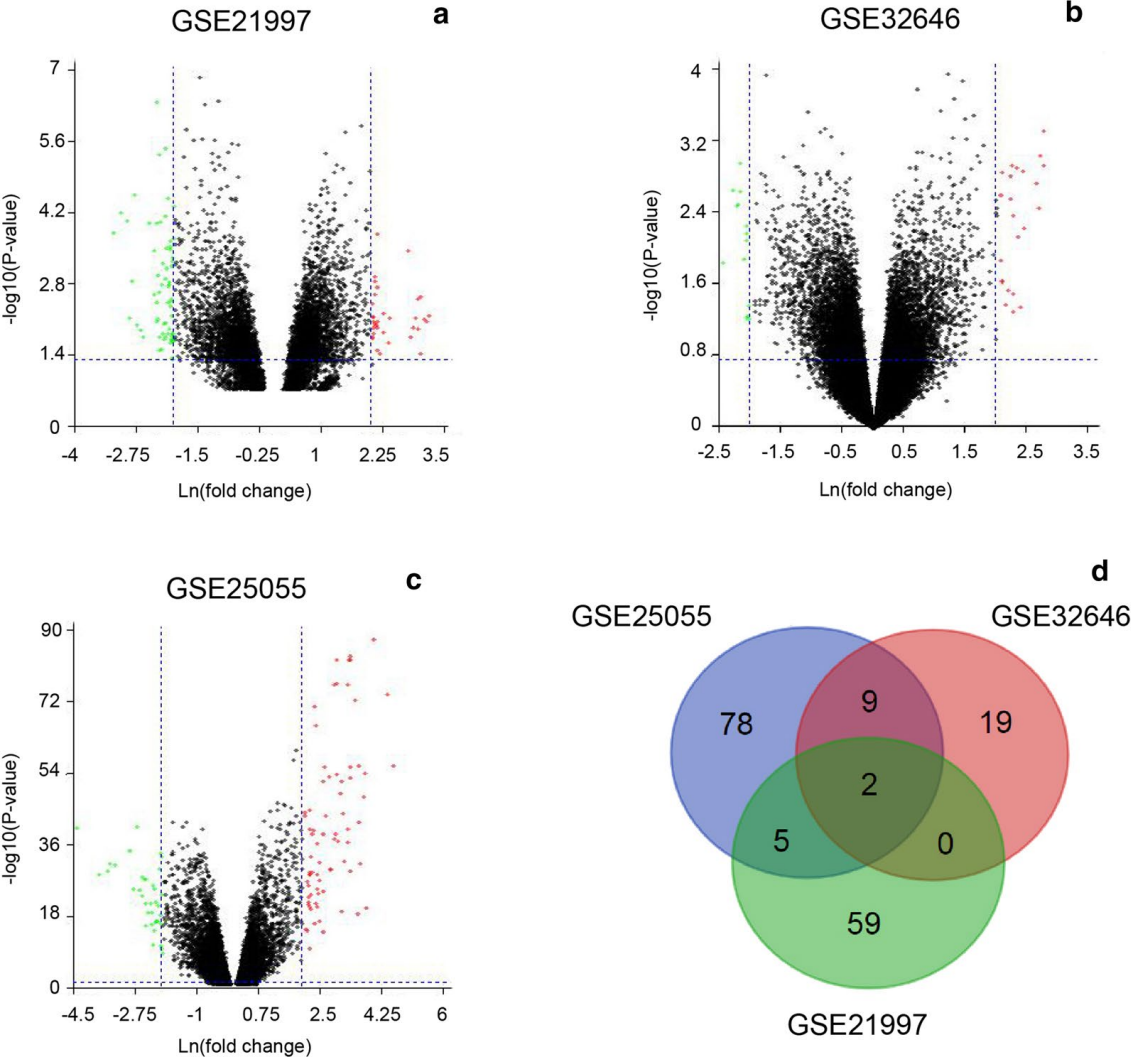
Identification of differentially expressed Genes. **a**–**c** Volcano plot of differentially expressed differentially expressed genes (DEGs) in GSE21997 (**a**), GSE32646 (**b**) and GSE25055 (**c**) DEGs with log2-Fold Change (log2FC) > 2 were shown in red; DEGs with log 2-Fold Change (log2FC) < − 2 were in green (p < 0.05). **d** Venn diagram reveals common DEGs among GSE21997, GSE32646 and GSE25055

We selected three GEO datasets (GSE21997, GSE32646 and GSE25055), which are genomic expression profiles for breast cancer subjects treated with neoadjuvant anthracycline and taxanes combination. Next, we compared gene expression profiles acquired by microarray analysis from subjects with or without residual tumor after neoadjuvant chemotherapy. A number of genes were identified as the potential predictors at the threshold of fold change ≥ 2 and *p*-value < 0.05. As shown in Fig. 1a–c, a total of 94 genes (61 up-regulated and 33 down-regulated genes) in GSE25055, 66 genes (19 up-regulated and 47 down-regulated genes) in GSE21997, and 30 genes (19 up-regulated and 11 down-regulated genes) in GSE32646 were filtered as differentially expressed Genes. The intersection identified a total of 2 differentially expressed genes, including FABP7 and ESR1, might be essential indicators of chemotherapeutic efficacy in breast cancer (Fig. 1d; Table 3).

**Table 3:** Differently expressed genes (DEGs) in three expression profiles

		Differently expressed genes (DEGs)
Three expression profiles	2	FABP7, ESR1
GSE25055 and GSE32646	9	TSPAN1, CPB1, CALML5, DNAJC12, TFF1, SCGB1D2, GFRA1, PGR, NPY1R
GSE25055 and GSE21997	5	LAMP3, GABRP, TFAP2B, EEF1A2, DNALI1

### Higher level FABP7 is correlated to better chemotherapeutic response, while higher level ESR1 is associated with poorer chemotherapeutic sensitivity

Next, we analyzed the association between either FABP7 or ESR1 mRNA level and chemotherapeutic response. It was found that mRNA level of FABP7 was considerably lower in residual tumor after NAC (GSE21997: *p *= 0.0264; GSE32646: *p *= 0.0075; GSE25055: *p *= 0.0004) (Additional file 1: Fig. S2A, S2C and S2E). On the contrary, the mRNA level of ESR1 was much higher in residual tumor after NAC (GSE21997: *p *= 0.0166; GSE32646: p < 0.0001; GSE25055: p < 0.0001) (Additional file 1: Fig. S2B, S2D and S2F). These results suggest that FABP7 might be associated with better chemotherapeutic response, while ESR1 might be related to poorer chemotherapeutic sensitivity.

### The expression of FABP7 is significantly lower in breast cancer than normal tissue samples

The analysis in ONCOMINE database demonstrated that the mRNA level of FABP7 was significantly lower in breast cancer than normal-tissue samples across a series of datasets in multiple cancer types (Additional file 1: Fig. S3A). The FABP7 mRNA expression in breast cancer samples was lower than that in normal tissues (fold changes were − 21.383, p = 2.66E−6 or − 8.265, *p *= 3.37E−9) (Additional file 1: Fig. S3B, C). On the contrary, ESR1 mRNA level was 4.032-fold (*p *= 3.37E−9) increased in breast cancer samples compared with normal tissue samples in Curtis breast statistics (Additional file 1: Fig. S3D, S3E). Similar trend (fold changes were 4.931, *p *= 7.58E−5) was found in The Cancer Genome Atlas (TCGA) breast statistics.

### FABP7 is particularly high expressed in ER-negative breast tumor and negatively associated with ESR1, GATA3 and FOXA1

**Fig. 2:**
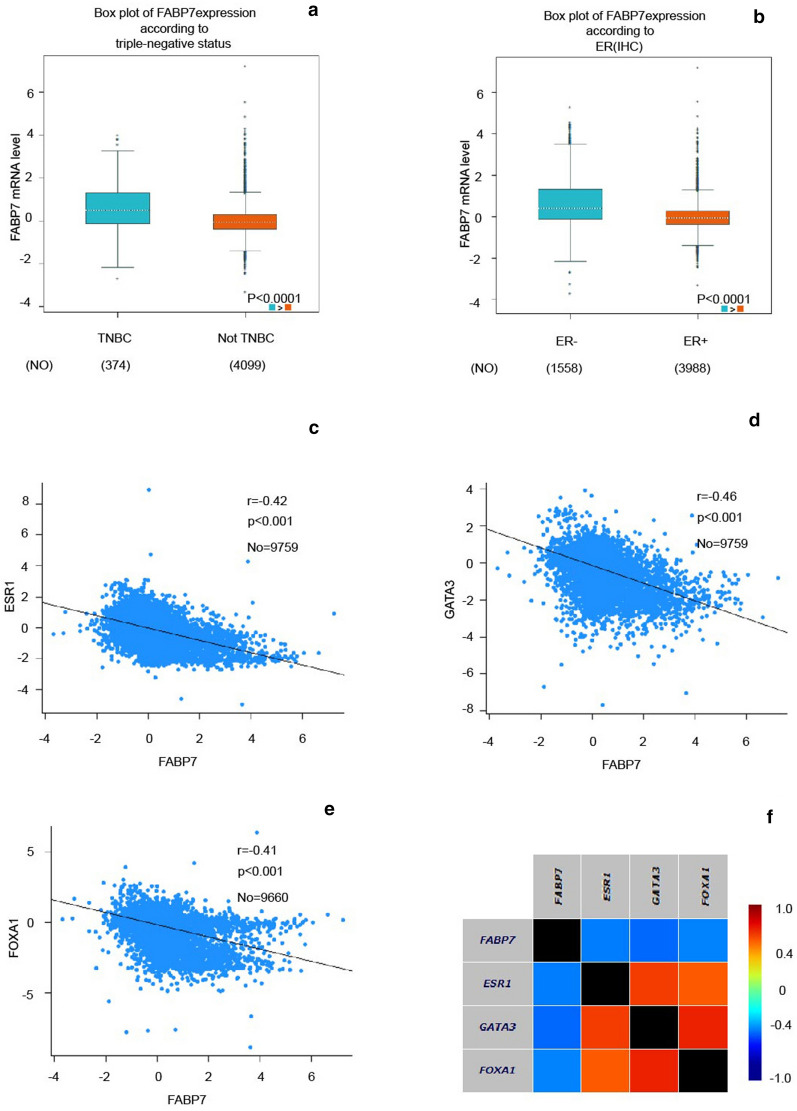
Higher expression of FABP7 correlated with low expression of ESR1, GATA3 and FoxA1. **a** The mRNA expression level of FABP7 in TNBC and not-TNBC patients. **b** The mRNA expression level of FABP7 in breast cancer patients with ER (−) and ER (+). **c** Gene correlation targeted analysis between FABP7 and ESR1. **d** Gene correlation targeted analysis between FABP7 and GATA3. **e** Gene correlation targeted analysis between FABP7 and FOXA1. **f** Correlation map for all patients among FABP7, ESR1, GATA3 and FOXA1

In bc-GenExMiner v4.0, the mRNA level of FABP7 in basal-like subtype tumors was significantly higher than non-basal-like subtype counterparts (Additional file 1: Fig. S4A). Similarly, the mRNA level of FABP7 was found significantly higher in triple-negative breast cancer (TNBC) than non-TNBC (Fig. 2a). Moreover, the highest FABP7 expression was observed in basal-like subtypes of breast cancer, while the lowest expression of FABP7 was found in the luminal subtypes (Additional file 1: Fig. S4B). The between-group comparisons were shown in Table 4. Higher FABP7 mRNA levels were found in patients with ER-negative than ER-positive tumors (Fig. 2b). Gene correlation targeted analysis indicated that higher expression of FABP7 in mRNA level was correlated to lower mRNA level of ESR1 (r = − 0.42, p < 0.001) (Fig. 2c), GATA3 (r = − 0.46, p < 0.001) (Fig. 2d) and FOXA1 (r = − 0.41, p < 0.001), which were typical epithelial biomarkers (Fig. 2e). Correlation maps for all patients among FABP7, ESR1, GATA3 and FOXA1 were showed (Fig. 2f). These results suggested that the mRNA level of FABP7 is particular higher in ER-negative breast tumor than other subtypes and was negatively associated with the mRNA levels of ESR1, GATA3 and FOXA1.

**Table 4:** The results of Dunnett–Tukey–Kramer’s test for pairwise comparison in different molecular subtypes of breast cancer

mRNA	Pairwise comparison of molecular subtypes	p value
FABP7	HER2 < Basal	< 0.0001
	LumA < Basal	< 0.0001
	LumA < HER2	< 0.0001
	LumB < Basal	< 0.0001
	LumB < HER2	< 0.0001
	Normal < Basal	< 0.0001
	Normal > LumA	< 0.0001
	Normal > LumB	< 0.0001
	LumB = LumA	> 0.1
	Normal = HER2	> 0.1

### DEGs were mainly involved in cell cycle and drug response

**Fig. 3:**
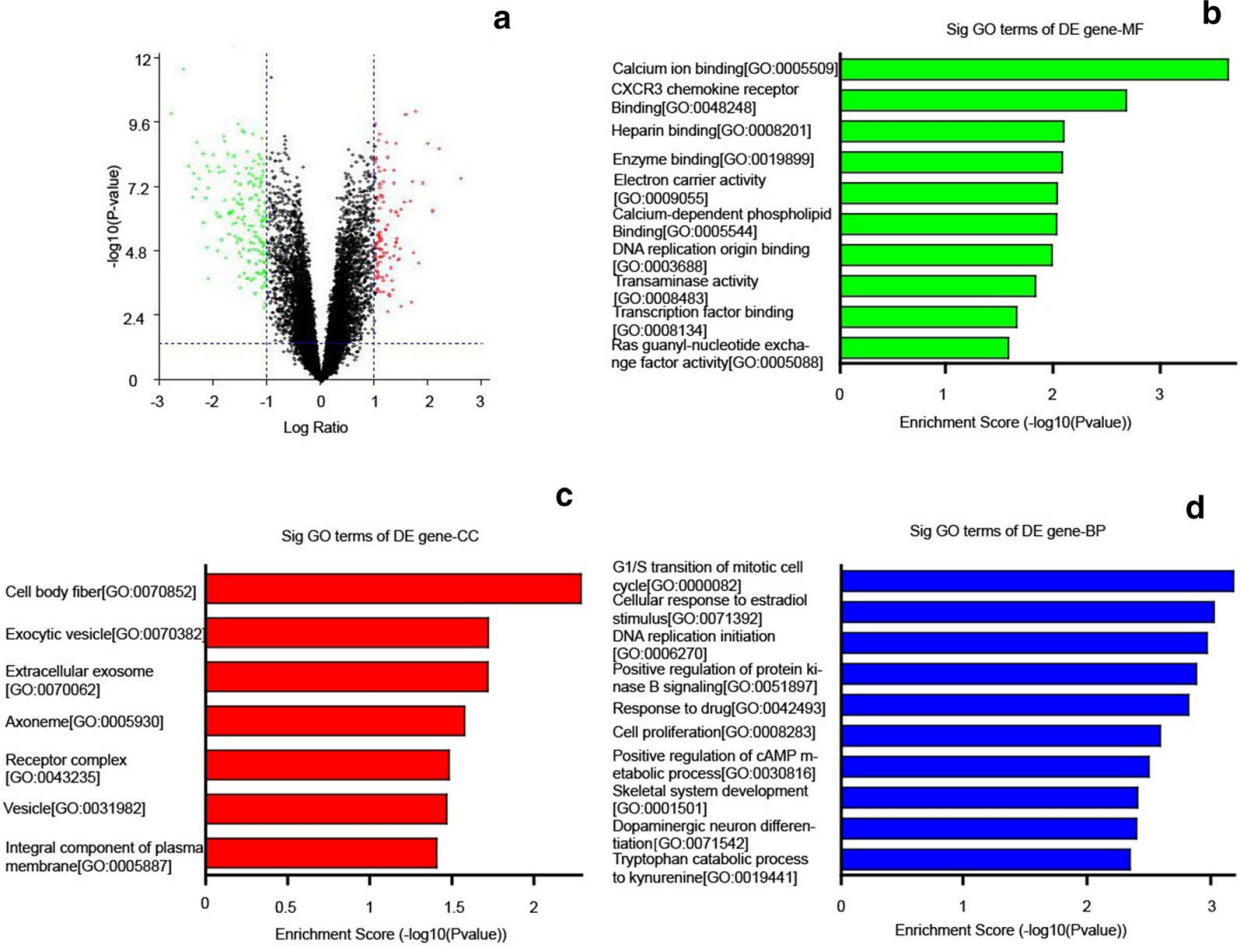
DEGs were mainly involved in cell cycle and drug response. **a** The Volcano plot of DEGs in breast cancer patients with/without FABP7 alterations. **b**–**d** Gene ontology enrichment analysis of DEGs. **b** Molecular function analysis. **c** Cellular component analysis. **d** Biological process analysis

The cBioPortal for Cancer Genomics database (TCGA, provisional) was used to analyze the DEGs (|log Ratio| ≥ 1 and p-value < 0.05) in breast cancer patients with or without FABP7 alterations, which was drawn with online tool (https://paolo.shinyapps.io/ShinyVolcanoPlot/) (Fig. 3a). The gene ontology (GO) enrichment analysis was conducted to identify the functional differences of DEGs and they were classed into three functional groups, including Molecular function (MF), Cellular Component (CC), and Biological Process (BP). The genes in the MF group were primarily enriched in heparin binding and calcium ion binding (Fig. 3b); the genes in the CC group were considerably enriched in cell body fiber, cell body fiber and extracellular exosome (Fig. 3c). The genes in the BP group were predominantly enriched in cell cycle regulation, cellular response to estradiol stimulus, as well as response to drug and cell proliferation (Fig. 3d). These results indicated that these DEGs might mainly be involved in cell cycle and drug response.

### Doxorubicin-resistant MDA-MB-231 cells lowly express FABP7 and tend to arrest cell cycle at G0/G1 phase compared with parental MDA-MB-231 cells

**Fig. 4:**
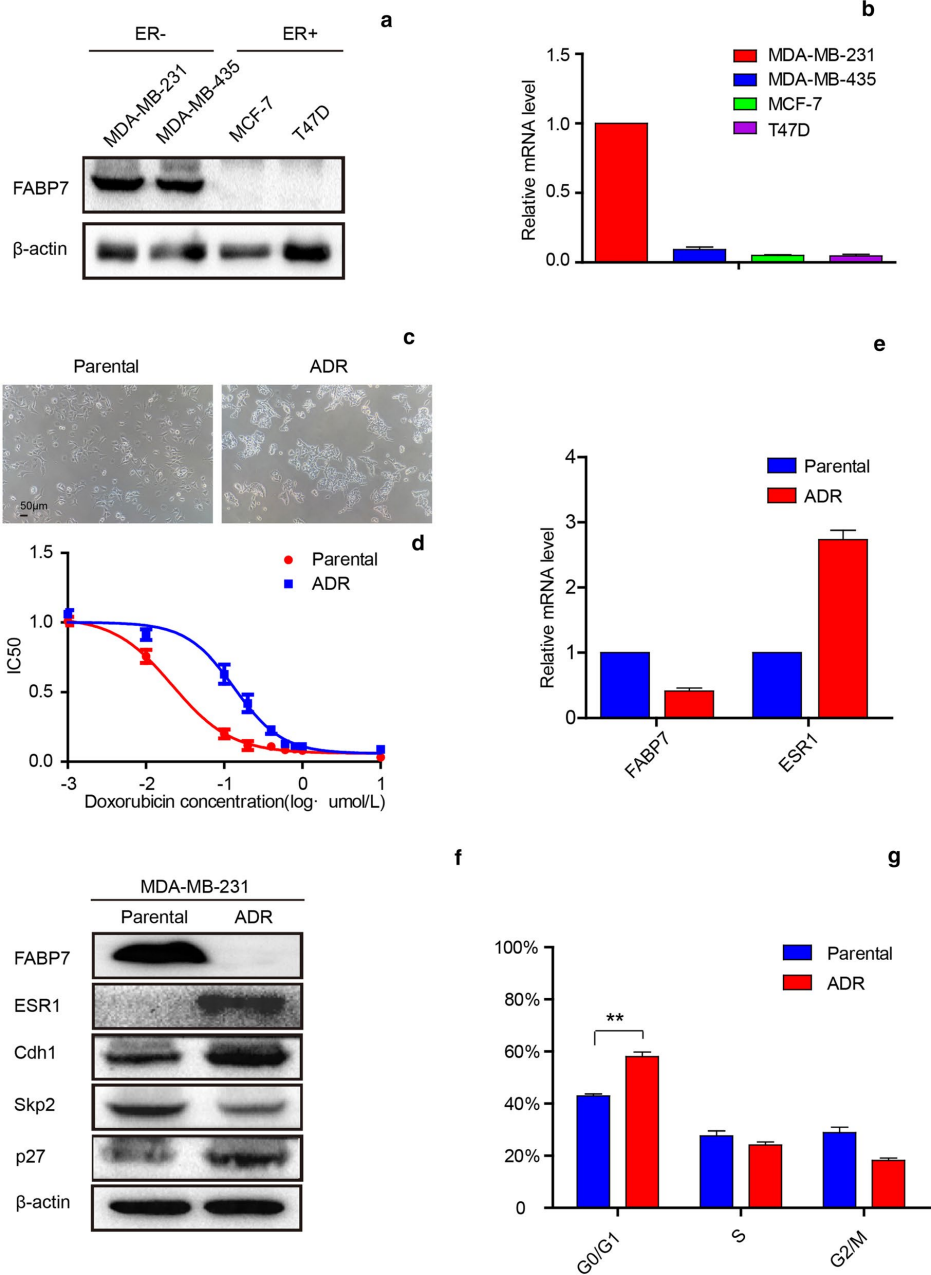
Compared with parental MDA-MB-231, MDA-MB-231-ADR lowly express FABP7 and tend to arrest cell cycle at G0/G1 phase. **a** The protein level of FABP7 in ER negative and ER positive group. **b** The mRNA levels of FABP7 in MDA-MB-231, MDA-MB-435, MCF-7, T47D breast cancer cells. **c** The morphology of MDA-MB-231 and MDA-MB-231-ADR cells. **d** The cell viability analysis of MDA-MB-231 and MDA-MB-231-ADR cells after treating with doxorubicin. **e** The mRNA levels of FABP7 and ESR1 in MDA-MB-231 and MDA-MB-231-ADR cells were measured by real-time PCR. **f** The protein level of FABP7, ESR1, Cdh1, Skp2, p27 were detected by Western blotting analysis in MDA-MB-231 and MDA-MB-231-ADR cells. **g** The different proportion of DNA in cell cycle in MDA-MB-231 and MDA-MB-231-ADR cells

To verify the bioinformatic analysis result, we performed western-blot assay and RT-PCR experiments. The results showed that FABP7 expressed significantly higher in ER-negative subgroups than ER-positive counterparts (Fig. 4a, b), which were consistent with our primary hypothesis. To examine whether the acquisition of doxorubicin resistance is accompanied by morphological changes, we observe the differences in cell morphology. As shown in Fig. 4c, MDA-MB-231-ADR cells exhibited rounded morphology and was more likely to cluster, compared to MDA-MB-231 cells, they were elongated spindle. We speculated that MDA-MB-231-ADR cells exhibit decreased mesenchymal phenotype but rather, the Epithelial phenotype.

To verify doxorubicin resistance in the MDA-MB-231-ADR cells, we treated both parental and MDA-MB-231-ADR cells with concentration gradient of doxorubicin and then determined the IC50 value with CCK8 assay (Fig. 4d). According to our data, the sensitivity to doxorubicin of MDA-MB-231-ADR cells was markedly lower as compared to the parental cells. Next, we performed RT-PCR and western blotting assays (Fig. 4e, f) to determine the expression of FABP7 and ESR1 in both mRNA and protein levels. We found that the expression in both protein and mRNA levels of FABP7 was significantly lower, which was negatively correlated with ESR1 in the MDA-MB-231-ADR cells than in the parental cells. Moreover, the FABP7 expression was also lower in MDA-MB-231 breast cancer cells treated with doxorubicin than in the control group (Additional file 1: Fig. S5).

Moreover, we examined whether the two cell lines (MDA-MB-231 and MDA-MB-231-ADR) influenced cell cycle. Therefore, we performed western blotting and flow cytometer assays to validate the expression level of relevant protein and DNA. As our dates shown, MDA-MB-231-ADR cells compared to parental cells, were more likely to arrest the cell cycle at G0/G1 phase (Fig. 4g). Next, we detected several vital proteins related to the G0/G1 phase, such as Cdh1, Skp2 and p27kip1. The Western blotting results showed that the expression of Cdh1 and p27kip1 were up-regulated while that of Skp2 was down-regulated in MDA-MB-231-ADR cells (Fig. 4f). These data suggest that FABP7 was negatively relative to ESR1 in doxorubicin resistance breast cancer cells. Furthermore, the sensitivity of cells to chemotherapy drugs is related to the cell cycle regulation. Therefore, we hypothesized that FABP7 could predict the drug sensitivity in breast cancer cells through regulating the cell cycle.

### FABP7 enhances drug sensitivity by promoting G1/S transition in cell cycle

**Fig. 5:**
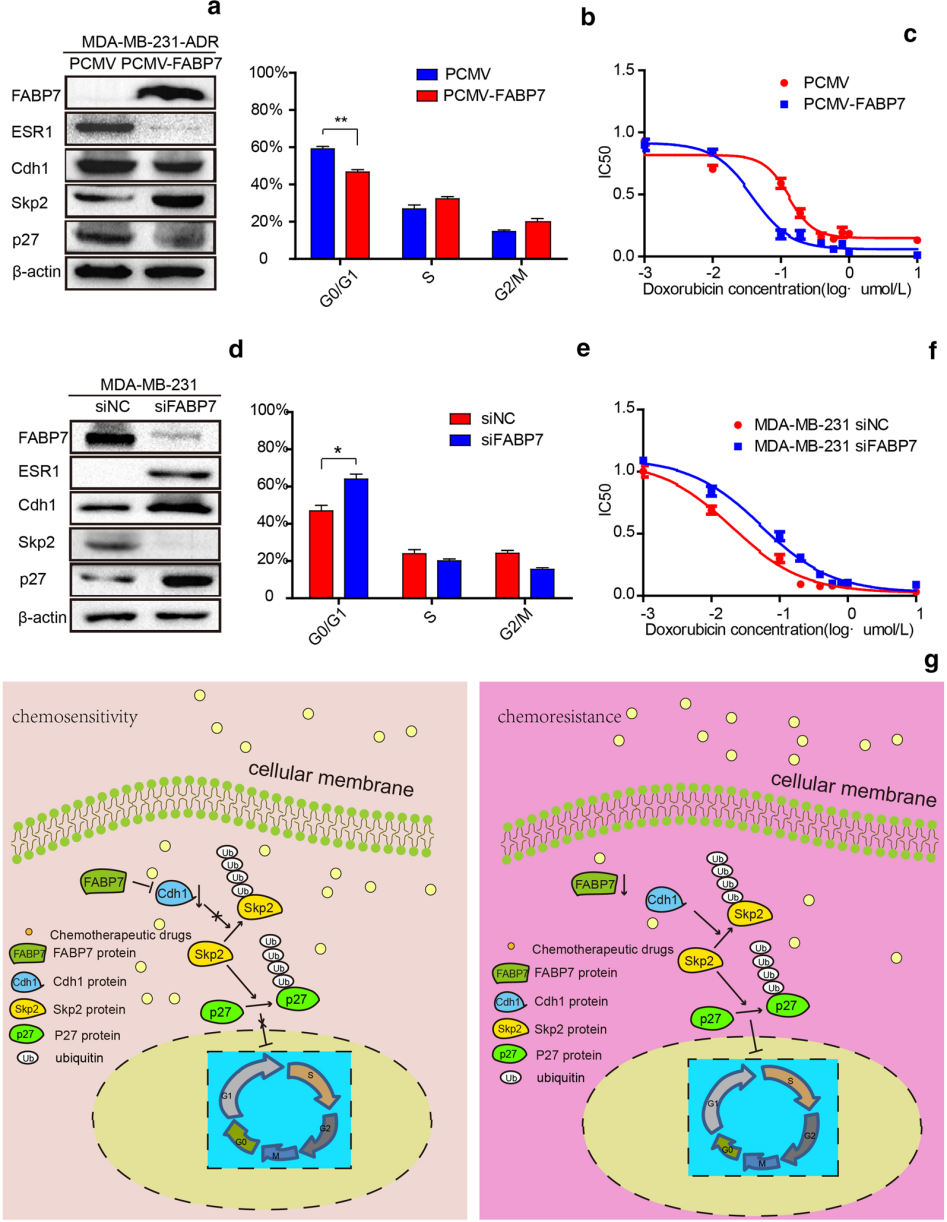
FABP7 enhances drug sensitivity by promoting G1/S transition in cell cycle. **a** The relative protein level of FABP7, ESR1, Cdh1, Skp2, p27 in overexpressed FABP7 MDA-MB-231-ADR and control group cells. **b** The effect of over expression FABP7 on cell cycle in MDA-MB-231-ADR cells. **c** The cell viability analysis of pCMV-FABP7 and pCMV in MDA-MB-231-ADR cells after treating with doxorubicin. **d** The relative protein level of FABP7, ESR1, Cdh1, Skp2, p27 in decreased expressed FABP7 MDA-MB-231 and control group cells. **e** The effect of silencing FABP7 on cell cycle in MDA-MB-231-ADR cells. **f** The cell viability analysis of siFABP7 and siNC in MDA-MB-231 cells after treating with doxorubicin. **g** The schematic representation of how FABP7 promotes proliferation and the relationship FABP7 and chemoresistance

We have found that FABP7 was negatively relative to ESR1 expression in MDA-MB-231-ADR cells. To further study the regulating relationship between FABP7 and ESR, we over-expressed FABP7 in MDA-MB-231-ADR cells by transient transfection of pCMV-FABP7 and we transfected small interfering RNA FABP7(siFABP7) or siNC in parental MDA-MB-231 cells. As shown in Additional file 1: Fig. S6 and Fig. 5a, overexpression of FABP7 could reduce the expression of ESR1 in MDA-MB-231-ADR in both mRNA and protein level. While silencing FABP7 in parental ER negative breast cancer cells, ESR1 expression increased (Fig. 5d).

Furthermore, to investigate whether the over-expression of FABP7 could make a difference in regulating cell cycle, we compared the pCMV-FABP7 and pCMV in MDA-MB-231-ADR cells with western blotting and flow cytometry assays. Our data showed that, compared with control MDA-MB-231-ADR, the high expression of FABP7 decreased the percentage of cells at G0/G1 phase and increased the percentage of cells at S and G2 phase (Fig. 5b). Considering the high expression of FABP7 might promote the transition of G1 to S phase, we examined several related proteins associated with cell cycle with western blotting assays. Our results showed that Cdh1, p27kip1 were decreased while the expression of Skp2 was up-regulated (Fig. 5a). When we silenced the expression of FABP7 in ER negative breast cancer MDA-MB-231 cells, we found exactly the opposite results (Fig. 5d), Cdh1 and p27kip1 increased expression, while the Skp2 was down-regulated.

Next, we determined whether the expression level of FABP7 correlated with doxorubicin resistance. We transfected pCMV-FABP7 into MDA-MB-231-ADR cells and siFABP7 into MDA-MB-231 cells. We subsequently measured IC50 value with CCK8 assay to determine their sensitivity to doxorubicin. Our data showed that over-expression of FABP7 could increase the sensitivity of MDA-MB-231-ADR cells to doxorubicin, while silencing FABP7 would decrease the sensitivity of MDA-MB-231 cells to doxorubicin (Fig. 5c, f). Taken together, these data suggest that overexpression of FABP7 increases doxorubicin sensitivity in MDA-MB-231-ADR cells. On the other hand, inhibition of endogenous FABP7 in MDA-MB-231 cells induces doxorubicin resistance. Besides, the expression level of FABP7 could affect cell cycle progress. Thus, FABP7 might enhance drug sensitivity by regulating the cell cycle process.

### Increased FABP7 was linked to longer recurrence-free survival (RFS) in BC subjects treated with adjuvant chemotherapy, particularly in those with ER-negative subtype of BC

**Fig. 6:**
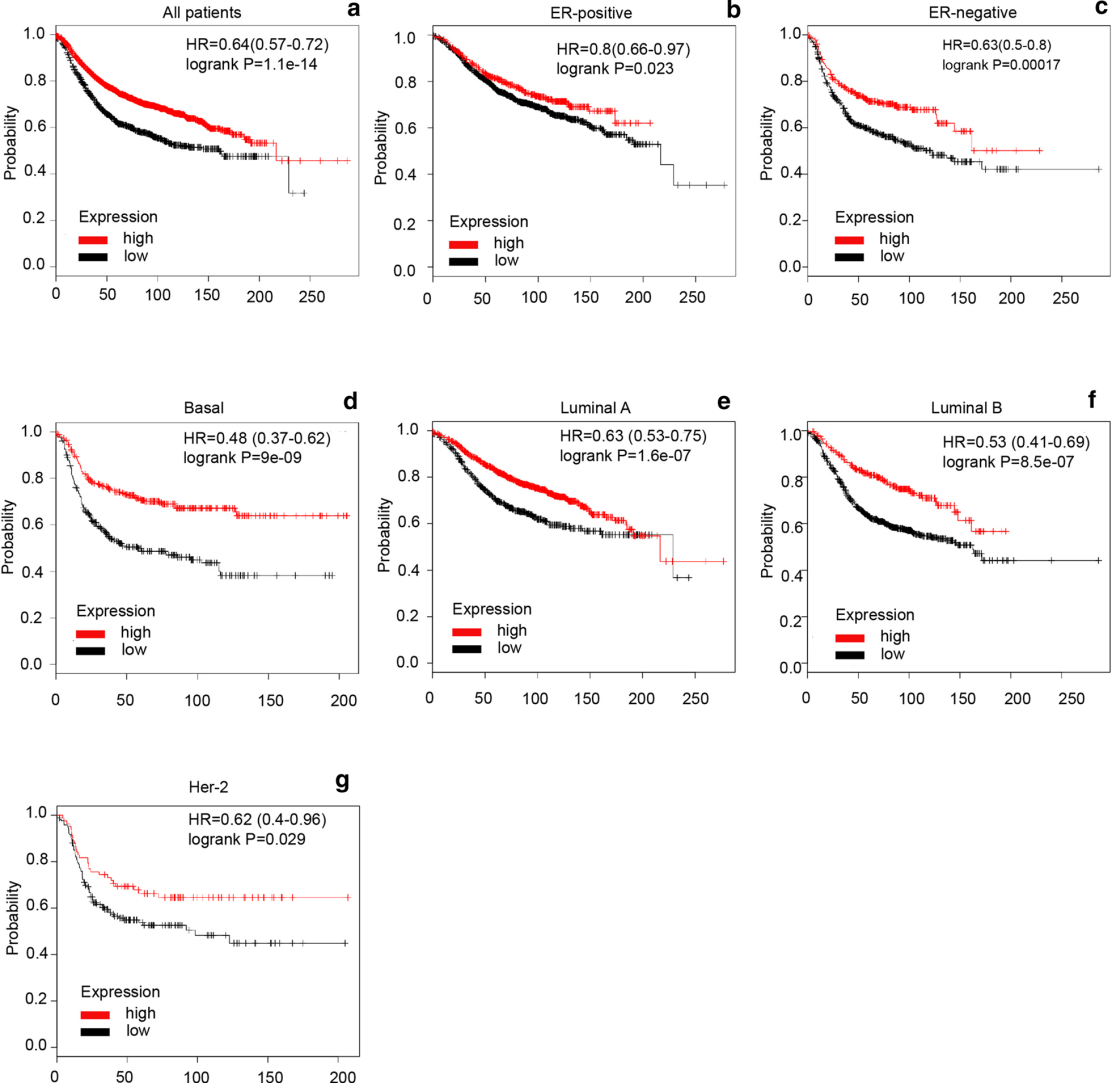
The prognostic value of FABP7 in breast cancer. **a** High mRNA level of FABP7 was associated with longer RFS in all BC patients. **b**, **c** High mRNA level of FABP7 was associated with longer RFS both in ER + (**b**), and ER− (**c**) BC patients. **d**–**g** The Kaplan–Meier plotter survival analysis showed that FABP7 mRNA expression was correlated to RFS in different subtypes of BC patients

Survival analysis demonstrated that higher mRNA level of FABP7 was closely linked to longer RFS in all BC subjects (HR = 0.64, *p *= 1.1E−14) (Fig. 6a). Subgroup analysis suggested that higher FABP7 mRNA level was significantly related to better RFS in subjects with ER-positive (HR = 0.8, *p *= 0.023) (Fig. 6b), ER-negative (HR = 0.63, p = 0.00017) (Fig. 6c), basal-like (HR = 0.48, *p *= 9e−09) (Fig. 6d), Luminal-A (HR = 0.63, *p *= 1.6e−07) (Fig. 6e), Luminal-B (HR = 0.53, *p *= 8.5e−07) (Fig. 6f) and Her-2 positive tumors (HR = 0.62, *p *= 0.029) (Fig. 6g). These results suggest that FABP7 is a strong predictor of favorable prognosis in patients with ER-negative breast cancer.

Moreover, higher expression of FABP7 in mRNA level was significantly correlated to better RFS in subjects who have received treatments including either chemotherapy (HR = 0.71, p = 0.015) (Additional file 1: Fig. S7A) or neoadjuvant chemotherapy (HR = 0.5, *p *= 0.022) (Additional file 1: Fig. S7D). Of noteworthy, higher mRNA level of FABP7 is linked to better RFS only in patients with ER-negative tumor treated with chemotherapy (Additional file 1: Fig. S7C) and adjuvant chemotherapy (Additional file 1: Fig. S7F), but not in ER-positive breast cancer patients (Additional file 1: Fig. S7B and S7E). These results indicate that elevated mRNA level of FABP7 predicts longer RFS in patients with ER-negative subtype of BC treated with chemotherapy or adjuvant chemotherapy.

## Discussion

Neoadjuvant chemotherapy (NAC) refers to administration of chemotherapeutic agents before tumor resection, with purpose of downstaging locally advanced breast cancer to an operable tumor, as well as eradicating occult distant metastases [16, 17]. However, a subset of patients might not respond to neoadjuvant chemotherapy, possibly due to intrinsic chemoresistance, resulting in compromise of the treatment efficacy and affecting the success of following surgery [18]. Identification of potential biomarkers to predict the response to NAC might help to guide the chemotherapeutic regimen selection.

In the present study, analysis of three genomic profiles from the GEO indicated that FABP7 and ESR1 were essential indicators of response to anthracycline and taxanes in breast cancer. It has been recognized that estrogen receptors alpha (ERα), encoded by ESR1, are positively expressed in about 65% of breast cancer subjects. A plethora of preclinical and clinical studies have demonstrated that positive ERα expression in breast cancer cells was associated with decreased sensitivity to chemotherapy [19]. Moreover, ERα contributed drug resistance partly through breast cancer resistance protein (BCRP) [20]. Our results corroborated the previous perspective on the association between ER expression and chemosensitivity, suggesting that ER-positive subtype of breast cancer is more insensitive or resistant to chemotherapy than ER-negative tumors.

Most intriguingly, we identified that FABP7 might be a novel biomarker to predict the response to neoadjuvant chemotherapy. FABP7, a family member of fatty acid binding proteins (FABPs), is recognized to facilitate the transportation of fatty acids (FAs) across a variety of cell organelles, regulating their metabolism and other physiological activities [21, 22]. Emerging studies have indicated that FABP7 was significantly involved in pathogenesis and progression of multiple cancer types and could be useful as a tumor marker [22, 23]. We found that FABP7, primarily high expressed in ER-negative breast tumor, was negatively associated with ESR1. The result confirmed the viewpoint of a study reported by Tang and colleagues, indicating that FABP7 overexpression exhibited a close link to triple-negative cases and the basal-like subtype of tumors [24]. Another study by Zhang H and colleagues also suggested that the proteins FABP7 was associated with the basal phenotype in human breast cancer [25]. Our results validated a higher frequency of FABP7 expression in basal-like/TNBC subtypes, as compared with other phenotypes or molecular subtypes of breast tumors.

Through DEGs and gene ontology (GO) enrichment analyses, we found that FABP7 were mainly involved in cell cycle and drug response. It is, therefore, hypothesized that the role of FABP7 in contribution to chemotherapeutic response could be partially mediated by influencing cell cycle progression.

In our study, we found that FABP7 was over-expressed in TNBC cells, while once acquired resistance to doxorubicin, the expression of FABP7 and ESR1 were reversed. The expression of FABP7 was negatively correlated with the expression of ESR1 in ADR cells. Moreover, over-expression of FABP7 could increase the doxorubicin sensitivity in ADR cells. Notably, we found that the low expression of FABP7 in ADR cells could lessen the cell proliferation activity and arrest the cell cycle at the G0/G1 phase in TNBC ADR cells. Then, flow cytometry experiment further demonstrated it. Theoretically, the low expression of FABP7 can activate Cdh1/Skp2/p27kip1 pathway, thus, leading to cell cycle arrest or quiescent. It is recognized that the p27kip1 gene is a tumor suppressor gene which inhibits the biological activity of cyclin–CDK complex, therefore, it can prevent cell transition from G1 phase to S phase. We also found that over-expression of FABP7 in ADR cells could promote the G1/S transition in cell cycle. Hence, FABP7 might accelerate the cell cycle by suppressing the activity of p27kip1, thus promoting the proliferation of breast cancer cells.

In addition, survival analysis from KM-plotter demonstrated that elevated FABP7 was associated with better RFS in BC patients treated with chemotherapy, especially in those with ER-negative subtype of BC. The result agrees with previous study by Zhang H and colleagues, which indicated that the overexpression of FABP7 was correlated to a better survival outcome in patients with breast cancer [25]. These findings imply that FABP7 is a favorable prognostic indicator for patients with breast cancer.

## Conclusions

Collectively, FABP7 highly expresses in and contributes to the chemosensitivity of ER-negative breast cancer, possibly via regulating the Cdh1/Skp2/p27kip1 pathway. Elevated FABP7 was closely linked to longer RFS in patients with ER-negative BC treated with chemotherapy or neoadjuvant chemotherapy. Future study of FABP7 as an independent biomarker or inclusion of FABP7 into a panel of genes in predicting the response to neoadjuvant chemotherapy for BC is warranted.

## Supplementary information

**Additional file 1: Fig. S1.** Flow chart of data preparation, processing, analysis, and validation. **Fig. S2.** The relationship between ESR1 or FABP7 mRNA level and chemoresistance. ESR1 expression in patients acquired by pCR and with residual tumor after receiving neoadjuvant treatment in GSE21997(A), GSE32646 (C) and GSE25055 (E). FABP7 expression in patients acquired by pCR and with residual tumor after receiving neoadjuvant treatment in GSE21997(B), GSE32646 (D) and GSE25055 (F). **Figure S3.** The FABP7 and ESR1 mRNA level in normal and cancer tissue. The mRNA expression of FABP7 and ESR1 in different cancer type. (B and C) Comparison of FABP7 mRNA expression in TCGA breast statistics; (D and E) Comparison of ESR1 mRNA expression in Curtis breast statistics (D) and TCGA breast statistics; (E). Box plots derived from gene expression data in ONCOMINE comparing expression of FABP7 and ESR1 in normal and BC tissue. The p-value was set up at 0.01 and fold change was defined as 2. **Figure S4.** The expression of FABP7 in different subtypes of breast cancer. (A) The expression of FABP7 in Basal-like and Not basal-like types. (B) The expression of FABP7 in several subtypes of breast cancer patients. **Figure S5.** The expression alteration of FABP7 in MDA-MB-231 breast cancer cells treated with doxorubicin. The western blot result of FABP7 expression in parental MDA-MB-231 breast cancer cells with or without doxorubicin. **Figure S6.** The RT-PCR assays reveal that the relative mRNA level of FABP7, ESR1 in overexpressed FABP7 MDA-MB-231-ADR and control group cells. **Figure S7.** Elevated FABP7 expression predicted better survival in breast cancer patients, especially in the ER(-), subgroups and BC patients received chemotherapy and neoadjuvant chemotherapy. (A–G) High mRNA level of FABP7 is associated with longer RFS in BC patients, who have received chemotherapy (A) and neoadjuvant chemotherapy (D), but not adjuvant chemotherapy (G). High mRNA level of FABP7 is associated with longer RFS in ER(-)BC patients, who have received chemotherapy (C) and adjuvant chemotherapy (F), but not in ER(+)BC patients. **Figure S8.** All protein expression level. Gray value measurement and statistical analysis of Western-blot in Figure 4 (A and B), Figure 5 (C and D).

## Data Availability

All data generated or analysed during this study are included in this published article and its supplementary information files.

## Abbreviations

FABP7: Fatty acid-binding protein 7
NAC: Neoadjuvant chemotherapy
RFS: Recurrence-free survival
ESR1: Estrogen receptor 1
CC: Cellular component
PCR: Pathologic complete response
TNBC: Triple negative breast cancer
BC: Breast cancer
ER: Estrogen receptor
MF: Molecular function
BP: Biological process
