# Supplementary material for: FABP7 is a potential biomarker to predict response to neoadjuvant chemotherapy for breast cancer
Source: Cancer Cell Int. 2020 Nov 23;20:562. doi: 10.1186/s12935-020-01656-3 (PMC7684949; doi:10.1186/s12935-020-01656-3)
Supplement: Supplementary file 1 — Additional file 1: Fig. S1. Flow chart of data preparation, processing, analysis, and validation. Fig. S2. The relationship between ESR1 or FABP7 mRNA level and chemoresistance. ESR1 expression in patients acquired by pCR and with residual tumor after receiving neoadjuvant treatment in GSE21997(A), GSE32646 (C) and GSE25055 (E). FABP7 expression in patients acquired by pCR and with residual tumor after receiving neoadjuvant treatment in GSE21997(B), GSE32646 (D) and GSE25055 (F). Figure S3. The FABP7 and ESR1 mRNA level in normal and cancer tissue. The mRNA expression of FABP7 and ESR1 in different cancer type. (B and C) Comparison of FABP7 mRNA expression in TCGA breast statistics; (D and E) Comparison of ESR1 mRNA expression in Curtis breast statistics (D) and TCGA breast statistics; (E). Box plots derived from gene expression data in ONCOMINE comparing expression of FABP7 and ESR1 in normal and BC tissue. The p-value was set up at 0.01 and fold change was defined as 2. Figure S4. The expression of FABP7 in different subtypes of breast cancer. (A) The expression of FABP7 in Basal-like and Not basal-like types. (B) The expression of FABP7 in several subtypes of breast cancer patients. Figure S5. The expression alteration of FABP7 in MDA-MB-231 breast cancer cells treated with doxorubicin. The western blot result of FABP7 expression in parental MDA-MB-231 breast cancer cells with or without doxorubicin. Figure S6. The RT-PCR assays reveal that the relative mRNA level of FABP7, ESR1 in overexpressed FABP7 MDA-MB-231-ADR and control group cells. Figure S7. Elevated FABP7 expression predicted better survival in breast cancer patients, especially in the ER(-), subgroups and BC patients received chemotherapy and neoadjuvant chemotherapy. (A–G) High mRNA level of FABP7 is associated with longer RFS in BC patients, who have received chemotherapy (A) and neoadjuvant chemotherapy (D), but not adjuvant chemotherapy (G). High mRNA level of FABP7 is associated with longer RFS i [file 12935_2020_1656_MOESM1_ESM.doc]

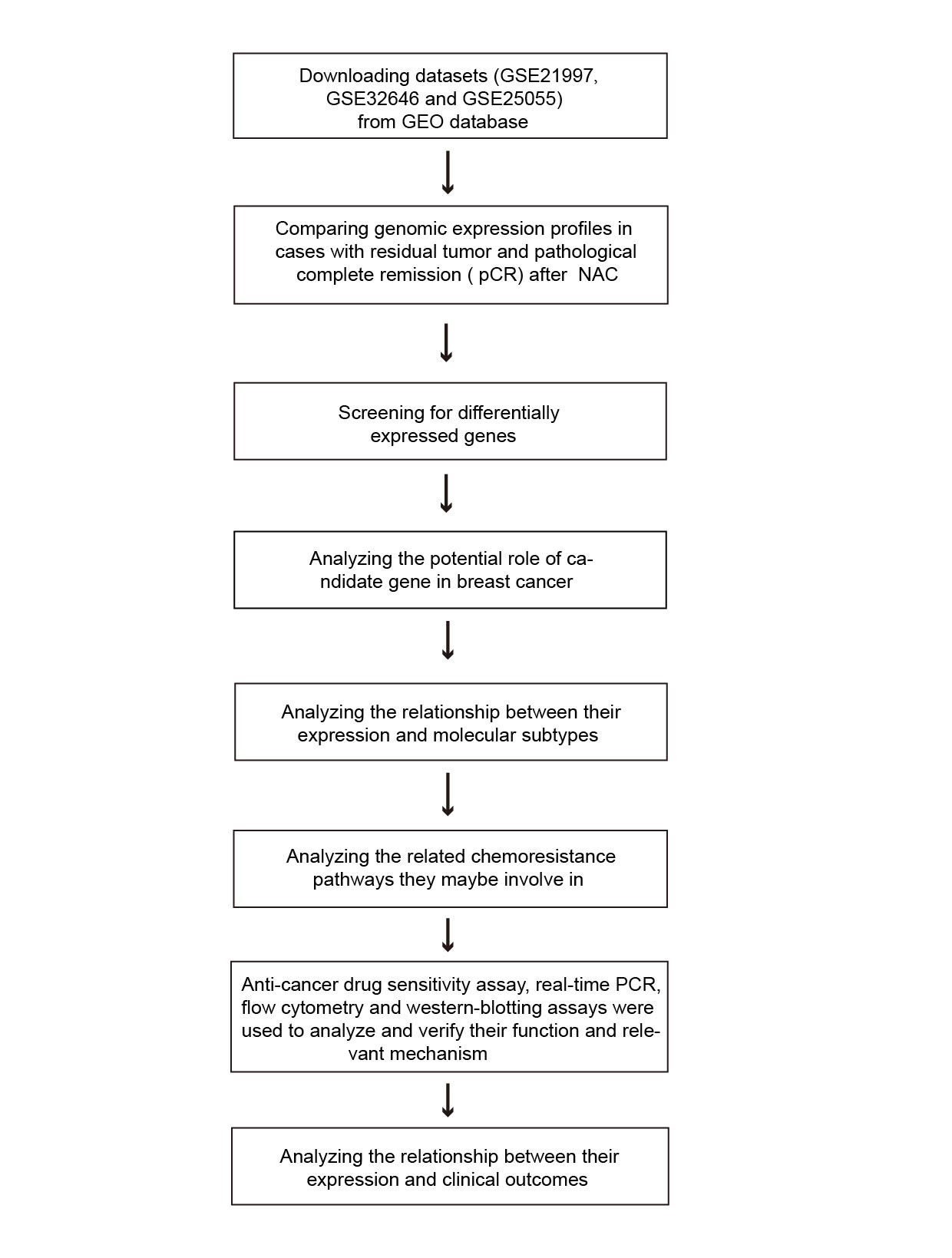


**Fig S1. Flow chart of data preparation, processing, analysis, and validation.**


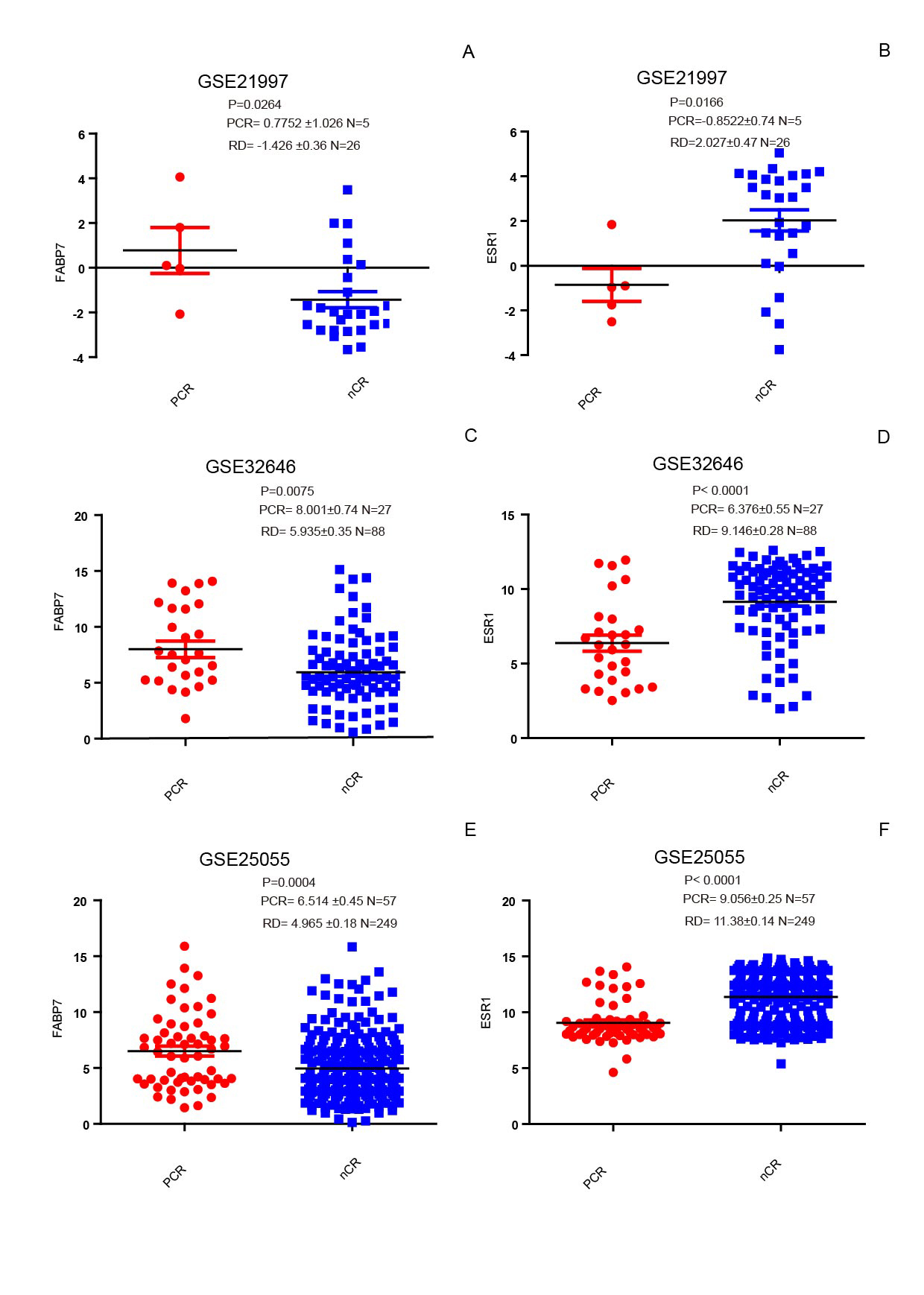


**Fig S2. The relationship between ESR1 or FABP7 mRNA level and chemoresistance.**

ESR1 expression in patients acquired by pCR and with residual tumor after receiving neoadjuvant treatment in GSE21997(A), GSE32646 (C) and GSE25055 (E). FABP7 expression in patients acquired by pCR and with residual tumor after receiving neoadjuvant treatment in GSE21997(B), GSE32646 (D) and GSE25055 (F).

**
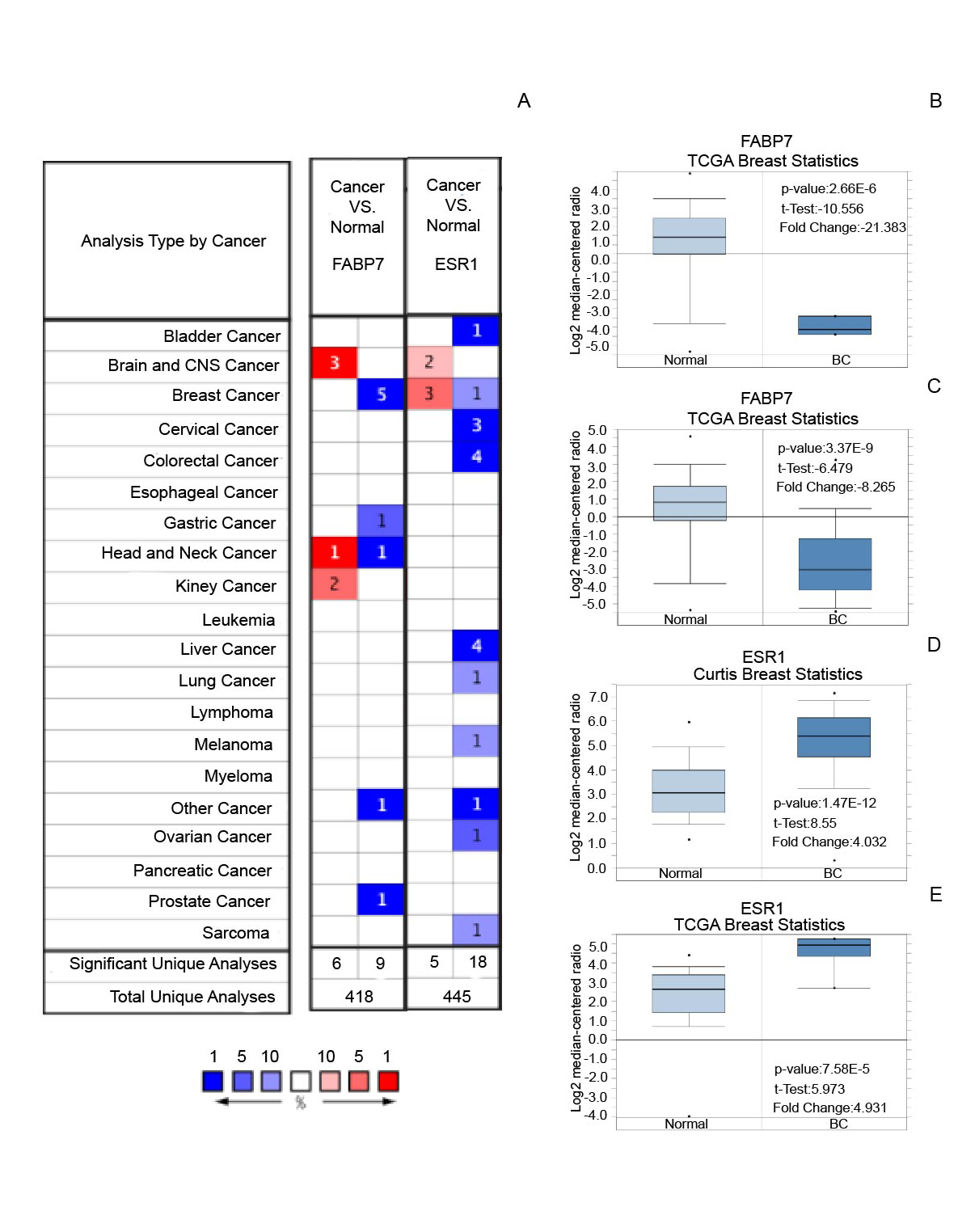
**

**Figure S3. The FABP7 and ESR1 mRNA level in normal and cancer tissue.**

The mRNA expression of FABP7 and ESR1 in different cancer type. (B and C) Comparison of FABP7 mRNA expression in TCGA breast statistics; (D and E) Comparison of ESR1 mRNA expression in Curtis breast statistics (D) and TCGA breast statistics; (E). Box plots derived from gene expression data in ONCOMINE comparing expression of FABP7 and ESR1 in normal and BC tissue. The p-value was set up at 0.01 and fold change was defined as 2.


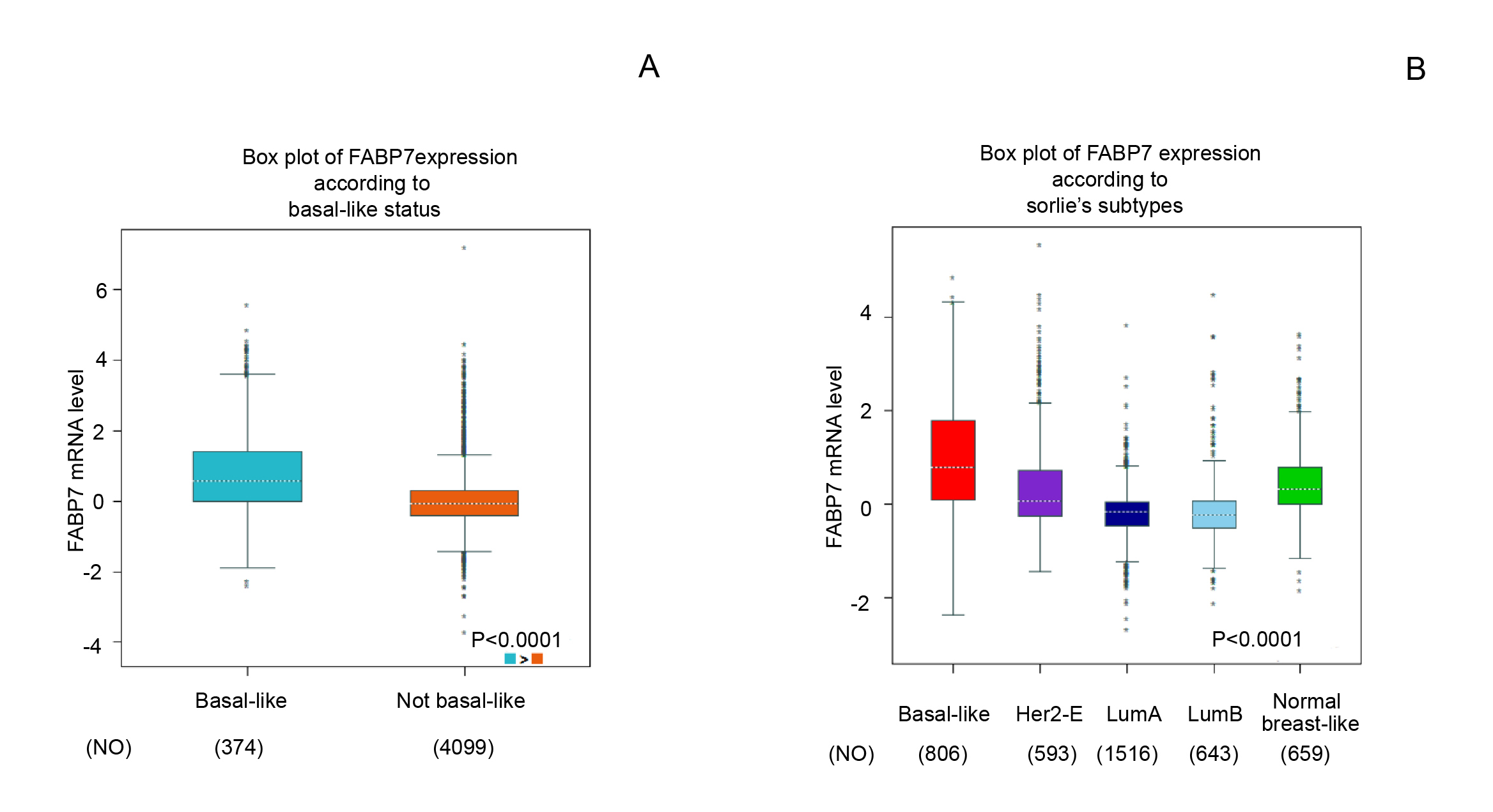


**Figure S4. The expression of FABP7 in different subtypes of breast cancer.**

(A) The expression of FABP7 in Basal-like and Not basal-like types . (B) The expression of FABP7 in several subtypes of breast cancer patients.


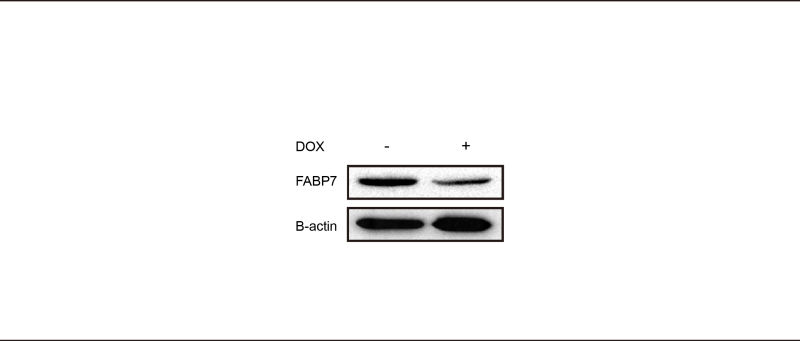


**Figure S5. The expression alteration of FABP7 in MDA-MB-231 breast cancer cells treated with doxorubicin .**

The western blot result of FABP7 expression in parental MDA-MB-231 breast cancer cells with or without doxorubicin.


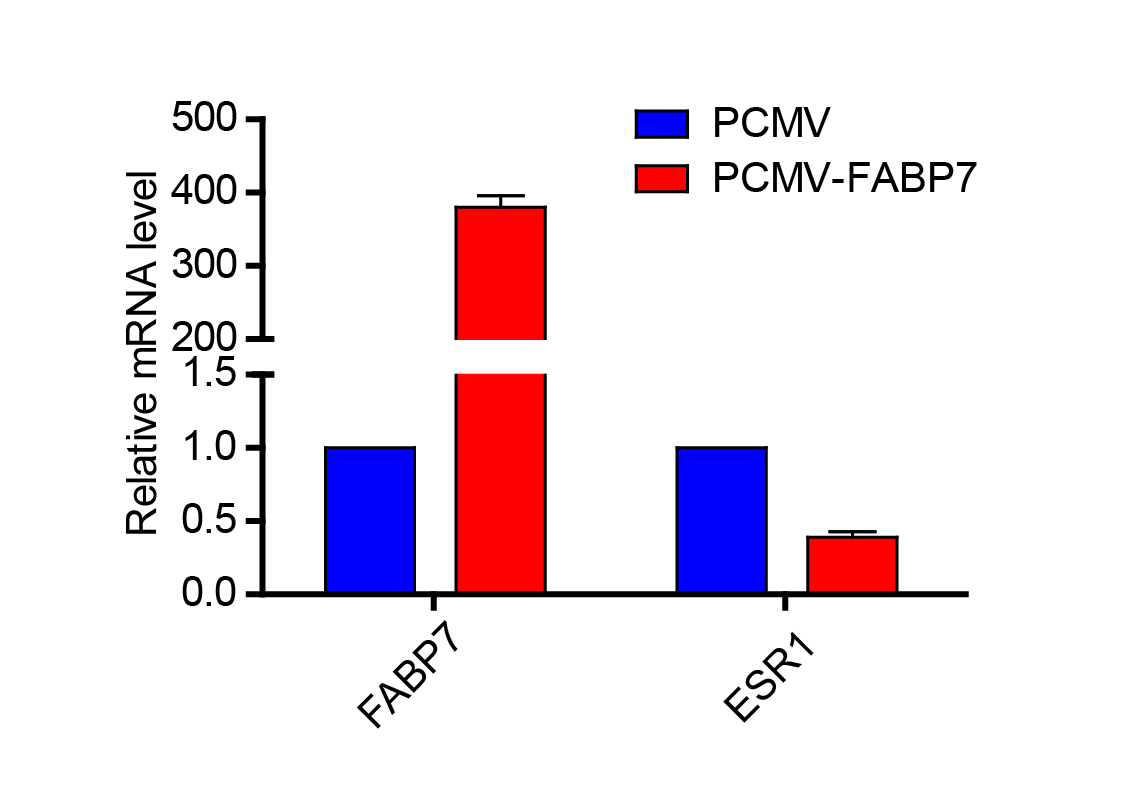


**Figure S6. The RT-PCR assays reveal that the relative mRNA level of FABP7, ESR1 in overexpressed FABP7 MDA-MB-231-ADR and control group cells.**


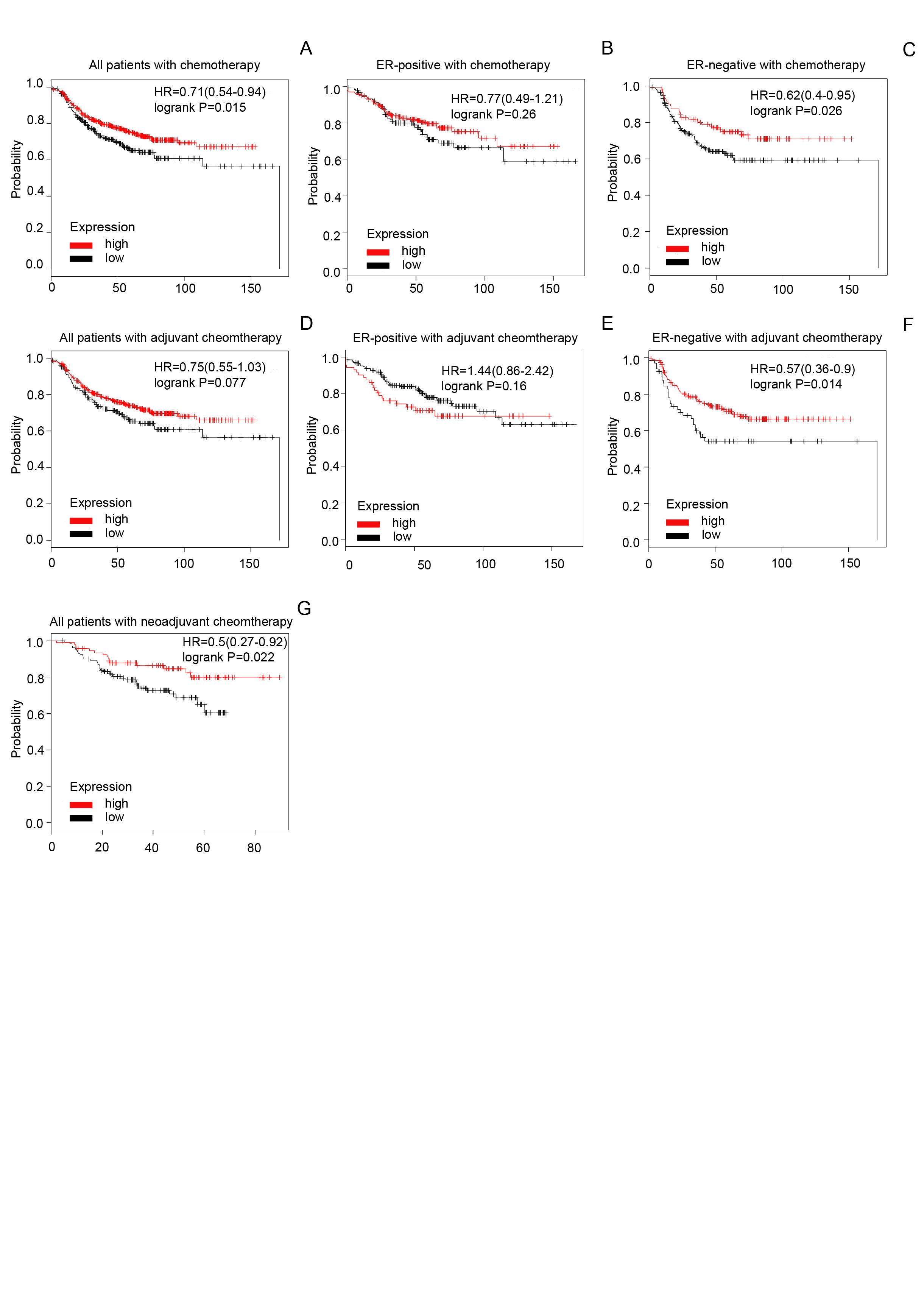


**Figure S7. Elevated FABP7 expression predicted better survival in breast cancer patients, especially in the ER(-),subgroups and BC patients received chemotherapy and neoadjuvant chemotherapy.**

(A-G)High mRNA level of FABP7 is associated with longer RFS in BC patients, who have received chemotherapy (A) and neoadjuvant chemotherapy (D), but not adjuvant chemotherapy (G). High mRNA level of FABP7 is associated with longer RFS in ER(-)BC patients, who have received chemotherapy (C) and adjuvant chemotherapy (F), but not in ER(+)BC patients.


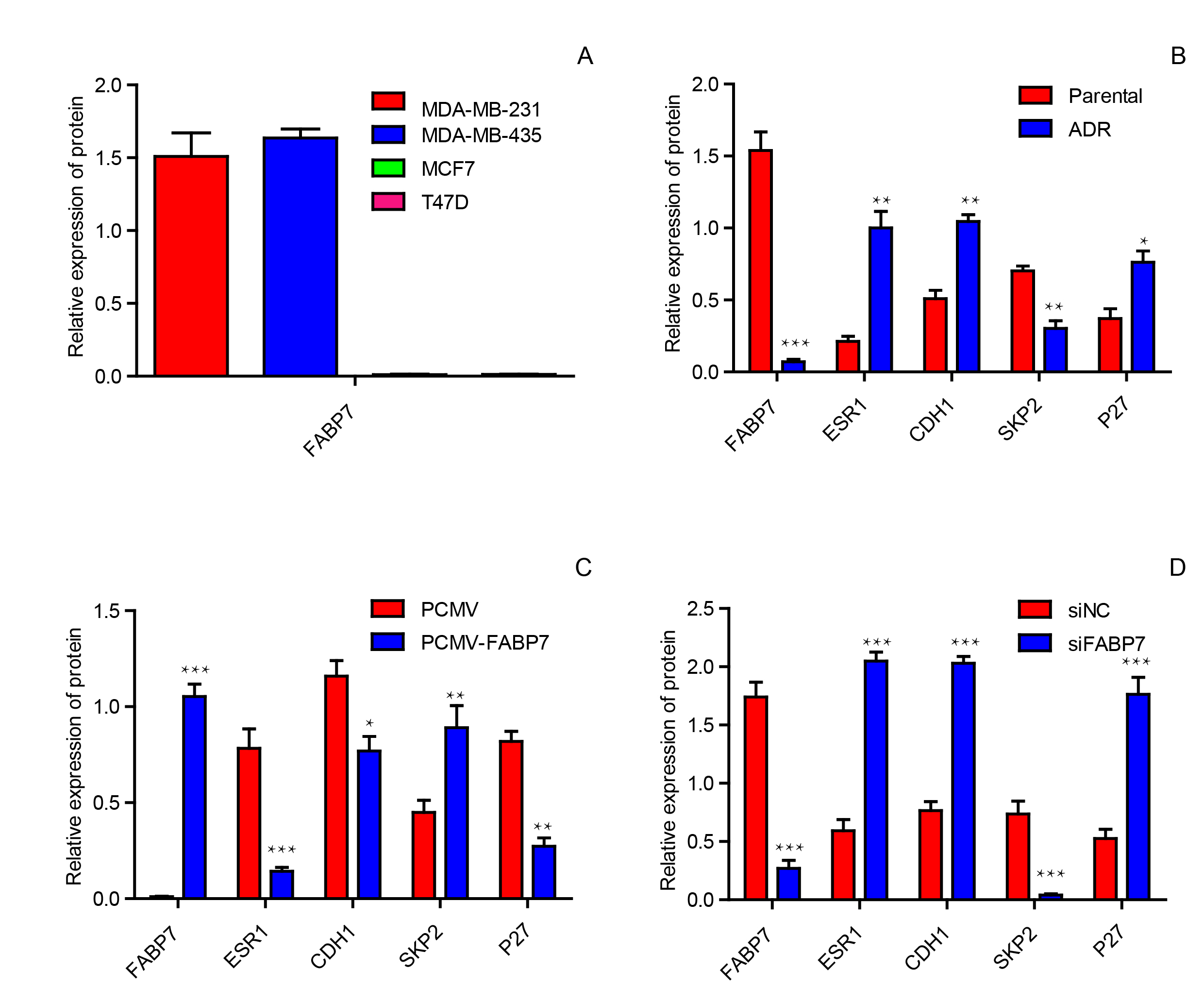


**Figure S8. All protein expression level.**

Gray value measurement and statistical analysis of Western-blot in Figure4（A and B）, Figure5（C and D）.
